# Knockdown of IL-1β Improves Hypoxia–ischemia Brain Associated with IL-6 Up-regulation in Cell and Animal Models

**DOI:** 10.1007/s12035-014-8764-z

**Published:** 2014-06-26

**Authors:** Sujuan Liu, Shengyun Zhu, Yu Zou, Tinghua Wang, Xuemei Fu

**Affiliations:** 1 Shenzhen Children’s Hospital, Shenzhen, Guangdong, 518038 China; 2 Institute of Neurological Disease, Translational Neuroscience Center, West China Hospital, Sichuan University, Chengdu, 610041 China

**Keywords:** Interleukin-1β, Hypoxia ischemia, Astrocyte, Neonatal

## Abstract

A study was conducted to investigate the effect of interleukin-1β (IL-1β) on hypoxia ischemia (HI) of cultured astrocyte and neonatal rat models and to explore the underlying molecular regulation mechanism. Primary rat astrocyte was exposed to hypoxia (2 % O_2_, 98 % N_2_) and cultured in serum-free medium for 6, 12, and 18 h to establish cell model of HI. Morphologic changes of astrocyte were monitored and gene expression change of IL-1β evaluated by real-time polymerase chain reaction (PCR). To establish the HI animal model, 3 days old postnatal Sprague–Dawley (SD) rats were treated with the right carotid artery ligation and were exposed to 8 % oxygen for 8, 16 and 24 h, respectively. Longa score scale, hematoxylin and eosin (HE) staining and water content were examined to assess neurologic function and morphology changes. IL-1β siRNA lentivirus (IL-1β-RNAi-LV) was injected into cerebral cortex motor area 2 days before HI and the interference efficiency examined by real-time PCR and Western blotting, respectively. Immunofluorescence staining of GFAP and IL-1β was performed to identify the location and interference effect of IL-1β, respectively. To further explore the potential mechanisms, the expression of inflammatory factors, including IL-6, IL-10 and tumor necrosis factor-alpha (TNF-α), was examined following IL-1β down-regulation. The size of soma astrocyte was increased greatly after 12 and 18 h of HI with IL-1β up-regulation. IL-1β knockdown by siRNA in vitro or by lentivirus in vivo can reverse cell swelling, brain edema and neurologic function deficiencies induced by HI. Lastly, interference of IL-1β remarkably increased IL-6 expression but not IL-10 and TNF-α. Therefore, down-regulation of IL-1β improves the deficiencies of neurologic function and morphology induced by HI, maybe closely associating with IL-6 regulation.

## Introduction

Hypoxia–ischemia (HI) insult of neonatal is a vital cause of perinatal brain injury, which may cause cerebral palsy, seizures, learning limitations and epilepsy [1–4]. The incidence of neonatal HI increases risk of mortality and lifelong morbidity in newborns, and reaches 2–6‰ live term births each year in Western countries [5, 6]. Currently, there is no effective therapy that could minimize brain disorder induced by HI in clinical. The treatment strategies are restricted due to the lack of knowledge regarding the related neuronal networks and the underlying mechanisms of neurodegeneration induced by HI.

Inflammation, as a complex intrinsic network of multiple subsets of immune cells, plays a pivotal role in HI pathogenesis. The phenomenon of apoptotic, swelling, necrotic cell deaths and excitotoxicity is prominent in the developing brain edema after HI [7–10]. However, inflammation is a protective response of the organism to remove the injurious events and to initiate the healing process. Interleukin-1β (IL-1β), a member of the interleukin 1 cytokine family that was produced by activated macrophages as a proprotein, is known to play a major role in the development of osteoarthritis, hypoxic ischemic encephalopathy and hepatic damage [11–13].

However, the exact role of IL-1β in brain edema with HI remains elusive. Recent studies have shown that inhibition of IL-1 expression by IL-1 receptor antagonist could attenuate dysfunction following HI [14]. IL-1β, interleukin-6 (IL-6) and tumor necrosis factor-alpha (TNF-α) in cerebrospinal fluid (CSF) were correlated with the severity of brain injury and can serve as the diagnosis indicators in infants who suffered from HI [12]. Additionally, intracerebral injection of IL-1β in adult mice activated nuclear factor-kappaB (NF-κB) signaling pathway and induced leukocytes infiltration into the brain tissue [15]. Although there is clear evidence that IL-1β plays a crucial role in HI, there is no sufficient evidence to identity the cause-and-effect relationship and potential regulation mechanism. We therefore studied the role of IL-1β in the HI astrocyte cell model and neonatal animal model to determine the underlying molecular mechanism.

## Methods

### Primary Culture of Astrocytes and HI Cell Model

For isolation of rat cerebral astrocytes, 3 days old neonatal rat pups were provided by Sichuan University according to Institutional Animal Care and Use Committee guidelines. They were deeply anesthetized by diethylether and euthanized by decapitation. Leaving behind the white matter, the cortex tissue was gently separated, and the meninges were gently peeled. Cortices were cutting into small pieces, dissociated into a cell suspension and plated in 75-cm^2^ tissue culture flasks (Corning). Tissue with modified DMEM/F12 culture media at a concentration of 1.5 × 10^6^ cells/ml was incubated in standard humidified 5 % CO_2_ at 37 °C for 72 h. Cells were maintained by feeding every 3 days with the fresh medium. After culturing cells for 7–9 days, flasks were shaken at 350 rpm for 6 h at 37 °C to separate the oligodendrocytes from the astrocytes. Those shaken flasks were exchanged with 10 ml of fresh medium and the shaking procedure was repeated for an additional 18 h to harvest purified astrocytes. The purity of astrocyte was evaluated with staining with glial fibrillary acidic protein (GFAP). Then cells were exposed to hypoxia (2 % O_2_, 98 % N_2_) and cultured in serum-free medium for 6, 12 and 18 h to establish cell model of HI. Morphologic changes of astrocyte following HI was observed under a phase contrast microscopy.

### Quantitative RT-PCR

Total RNA was extracted from primary rat astrocytes using Trizol reagent (Invitrogen) and reversed transcribed to cDNA with the RevertAid™ First Strand cDNA Synthesis kit (Invitrogen) according to the manufacturer’s protocol. QRT-PCR for five genes was then performed to examine the expression of mRNA. The primer sequences were listed as follows: IL-1β: forward, 5′-GAGCTGAAAGCTCTCCACCT-3′; reverse, 5′-TTCCATCTTCTTCTTTGGGT-3′; IL-6: 5′-AGAAGACCAGAGCAGATTTT-3′; reverse, 5′-GAGAAAAGAGTTGTGCAATG-3′; IL-10: 5′-CAGAAATCAAGGAGCATTTG-3′f; reverse, 5′-CTGCTCCACTGCCTTGCTTT-3′; TNF-α: 5′-GCCCACGTCGTAGCAA-3′; reverse, 5′-GTCTTTGAGATCCATGCCAT-3′; β-actin: 5′-GAAGATCAAGATCATTGCTCCT-3′; reverse, 5′-TACTCCTGCTTGCTGATCCA-3′; Gene expression quantitation was carried out in a DNA thermal cycler (ABI 7300) according to the following standard protocol: a denaturation step of 95 °C for 2 min; 40 cycles of 95 °C for 15 s, annealing for 20 s, and annealing temperature at 60 °C for 40 s. The housekeeping gene β-actin was used as an endogenous control to normalize mRNA content for each sample, and non-template control was as negative controls for samples of multiple wells. The mRNA level was relatively quantified by using the 2^−△△Ct^ method.

### IL1-β siRNA

Not-targeting and targeting siRNA were obtained from Ruibo Company Guangzhou, China. Three 19-nucleotide sequences were designed corresponding to the IL-1β reference sequence (NCBI, NM_031512.2) to specific silencing of IL-1β. The sequence of those fragments was as follows: F1, CCAAGTCCGTCTTCTACAT; F2, CAGGTGCACTTTACGAGTA; F3, CAGCATGAATCCAGCTCGA. Non-targeting siRNA was constructed using a 19-nucleotide sequence which is no homology to any mammalian gene sequence as negative control.

### siRNA Transfection

To select the one with maximum interference efficiency of fragments of IL-1βsiRNA vector and the most optimized time point of transfection, cerebral astrocytes were transfected by three candidate target fragments of IL-1βsiRNA. There were four groups in this experiment as following: normal group, tansfection reagent group, negative control group and IL-1βsiRNA group. Cells were incubated with transfection mix (4 μl IL-1βsiRNA, 3 μl transfection reagent and 100 μl transfection buffer) for 2, 4 and 7 days according to the manufacturer’s instructions, respectively. The inhibition rate of three fragments and the best time point of transfection were validated by a quantitative real-time RT-PCR as described above.

Then the siRNA of highest inhibition rate was transfected into astrocytes of HI to explore the function of IL-1β. And the morphologic changes of astrocyte after IL-1βsiRNA treatment was observed and collected by a phase contrast microscopy. Non-targeting siRNA was transfected into astrocytes as negative control.

### IL-1β siRNA Lentivirus

The sequence information of the highest inhibition rate of IL-1βsiRNA was provided to GeneCopoeia Company (GuangZhou, China) to construct IL-1β siRNA expression vector. Briefly, IL-1β siRNA lentivirus ((IL-1β-RNAi-LV)) was produced by co-transfected IL-1β expression vector and viral packaging system (gag, pol and env) into 293 T cells according to the manufacturer’s protocol. Forty-eight hours after transfection, virus medium was harvested, filtered through a 0.45-mm cellulose acetate filter, purified and concentrated by concentration solution (GeneCopoeia Company). Finally, the IL-1β-RNAi-LV was frozen at -80 °C for following experiment.

### HI Rat Model and IL-1β-RNAi-LV Injection

Three-day-old SD rat pups (weighing 8–9 g), provided by the Animal Experimental Center of Sichuan University, were employed in this study. All animal procedures used were approved by University Committee on Animal Use and Care. Rat pups were randomly divided into four groups showed in Table 1. Briefly, the pups were kept under inhalation diethyl ether anesthesia during the entire procedure, and temperature of scalp and body was maintained at 37 °C by a homoisothermy bench. Following 0.5 cm skin incision in the midline of the neck, and the right common carotid artery (CCA) was permanently ligatured with 5–0 silk. Following surgery, rats were returned to their mother for recovery and feeding for 30 min. Then the pups were taken into an airtight chamber maintaining the ambient temperature inside the chamber at a constant 37 °C for hypoxia (8 % O_2_, 92 % N_2_) for 8, 16 and 24 h, respectively. The sham group only underwent a neck dissection and put the silk around carotid artery, but not ligated; the pups in HI group only occlude the CCA; IL-1β-RNAi-LV (4 μl) was injected into cerebral cortex motor area of right hemisphere in IL-1β-RNAi-LV group and no-targeting lentivirus injection was used as a negative control group.

**Table 1:** Animal grouping

Animal grouping	8 h	16 h	24 h
Sham group (Sham)	*n* = 12	*n* = 12	*n* =12
Hypoxia ischemia group (HI)	*n* = 12	*n* = 12	*n* =12
Hypoxia ischemia with no-targeting lentivirus (Negative Control)	*n* = 12	*n* = 12	*n* =12
Hypoxia ischemia with IL-1β-targeting lentivirus (IL-1β-RNAi-LV )	*n* = 12	*n* = 12	*n* =12

### Behavioral Analyses

The blinded Longa scale score test for neural impairment was performed before surgery and at different time points (8, 16 and 24 h) after HI surgery. There were five scales in this test as following. A score of 0 indicates no neurologic deficit; a score of 1 failing to extend left forepaw fully, indicating a mild focal neurologic deficit; a score of 2 circling to the left, indicating a moderate focal neurologic deficit; a score of 3 indicates a severe focal deficit with falling to the left; a score of 4 indicates a depressed level of consciousness and failure to walk spontaneously.

### Brain Water Content

For sham and HI groups, the brains of rat pups (*n* = 6) were used to measure the water content. The wet weight was measured first, and then the dry weight of tissue was measured after the tissue dried at 100 °C for 24 h. Brain water content was caculated by next forum: (as a percentage) = (wet weight − dry weight)/wet weight × 100 %.

### Hematoxylin and Eosin Staining

After anesthesia, rat pups were perfused through the ascending aorta with 5 ml of 0.1 mol/l phosphate-buffered saline (PBS; pH 7.2–7.3, room temperature), followed by 5 ml of 4 % paraformaldehyde in PBS. Tissues were paraffin-embedded after dehydration, serial horizontal sections (4 μm thickness) were stained with hematoxylin and eosin (HE) to examine the morphology changes of brain. Microscopic image was acquired by Leica AF6000 microscope.

### Immunofluorescence Staining

To examine the changes of astrocyte swelling, the location and expression of IL-1β following IL-1β-RNAi-LV injection, comparative analysis of immunofluorescence staining was performed in the brain tissue section. Briefly, the brain was removed and postfixed in 4 % paraformaldehyde solution for 24 h followed by gradient of dehydration in 10 %, 20 %, 30 % of sugar solution for 24 h successively. Tissues were OCT-embedded after dehydration, and cut vertically through injection point with 10 μm thickness by freezing microtome (Leica CM1900; Wetzlar, Hesse, Germany). The sections were incubated with rabbit anti-GFAP antibody (1:100; Zhongshan Jinqiao, China) together with mouse anti-IL-1β antibody (1:100; Abcam, Cambridge, MA, USA) at 4 °C overnight, and then followed by fluorescence secondary antibodies, including CY3 anti-rabbit IgG (red), Alexa Fluor 488 anti-mouse IgG (green) and Alexa Fluor 488 anti-rabbit IgG (green). Control experiments were carried out by replacing the primary antibodies with normal serum. Section image was collected using a confocal microscope (Zeiss LSM).

### Western Blotting Analysis

To examine whether the protein expression of IL-1β was reduced after IL-1β-RNAi-LV injection, brain samples were removed and protein extracted for western blotting experiment. Protein (80 μg) was separated by SDS-PAGE. After electrophoresis for 120 min at 75 V, the proteins were transferred to PVDF membranes (Millipore, Billerica, MA, USA). The membranes were then blocked in 5 % nonfat milk in PBST for 120 min at room temperature and incubated with primary antibody of IL-1β (Abcam), diluted 1:1,000 in 5 % BSA overnight at 4 °C. After incubation with the primary antibody, the membranes were washed in three changes of PBST and incubated for 2 h with secondary antibody (goat anti-rabbit IgG; ZSGB-BIO, China) diluted at 1:5,000. Finally, membranes were repeatedly rinsed in TBST three times and the immune complexes were revealed using Alpha Innotech (Bio-Rad) with ECL.

### Statistical Analysis

The statistical significance between groups was determined on the basis of one-way analysis of variance test (ANOVA) with SPSS17.0. Statistical significance was defined at *P* < 0.05. All data are expressed as mean ± SEM.

## Results

### Astrocytes Swelling and Inflammatory Factors Up-regulation Following HI

**Fig. 1:**
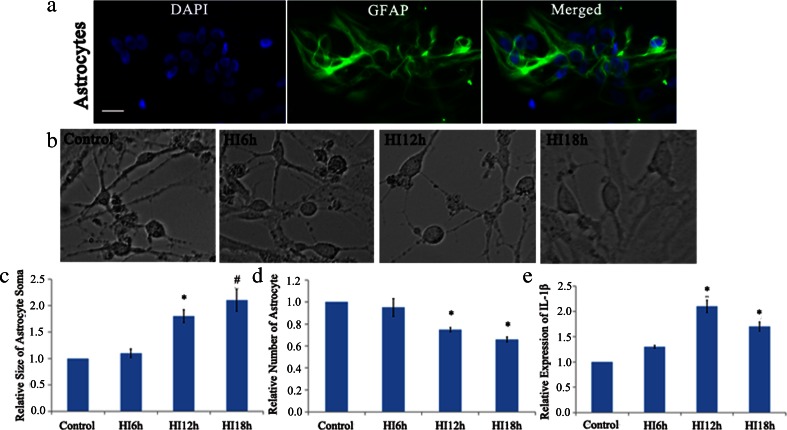
Effect of HI on astrocyte and IL-1β expression in vitro. The astrocytes could be identified by cytoplasmic localization of GFAP (*green*) immunofluorescent staining (**a**). Morphologic analysis showed HI can cause cell swelling, and the size of soma was significantly increased starting at 12 h after HI when compared with the control (**b**, **c**). Moreover, the number of cells was significantly reduced at 12 and 18 h after HI (**b**, **d**). Gene expression quantification showed IL-1β was significantly increased after HI when compared with that of normoxia group (**e**). Bar = 20 μm, shown in **a**. *P < 0.05, ^#^ *P* < 0.01, compared with control group

After purification of primary culture of cerebral astrocyte by mechanical shaking, GFAP, a specific maker of astrocyte, was used to stain for identification of the astrocyte. The result showed GFAP expressed in cytoplasm and emitted green fluorescence surrounding the blue nuclein (Fig. 1a). Those GFAP-positive cells were identified as astrocytes.

The images of astrocyte following HI were collected by a phase contrast microscopy. Compared with normoxia group, the size soma of astrocyte was significantly increased starting at 12 h after HI, suggesting that cell swelling was induced by HI (Fig. 1b,c). However, the number of astrocytes was significantly decreased in HI12h and HI18h (*P* < 0.05 vs. control; Fig. 1b,d), while there was no significant difference between the 6 h HI and normoxia groups. Therefore, 12 h of HI was used in the following studies (Fig. 1b–d).

PCR results showed the mRNA of IL-1β was significantly increased after HI treatment for 12 and 18 h when compared with that in normoxia group (Fig. 1e).

### The Most Effective siRNA Fragment and the Optimal Time Point of Transfection Selection

**Fig. 2:**
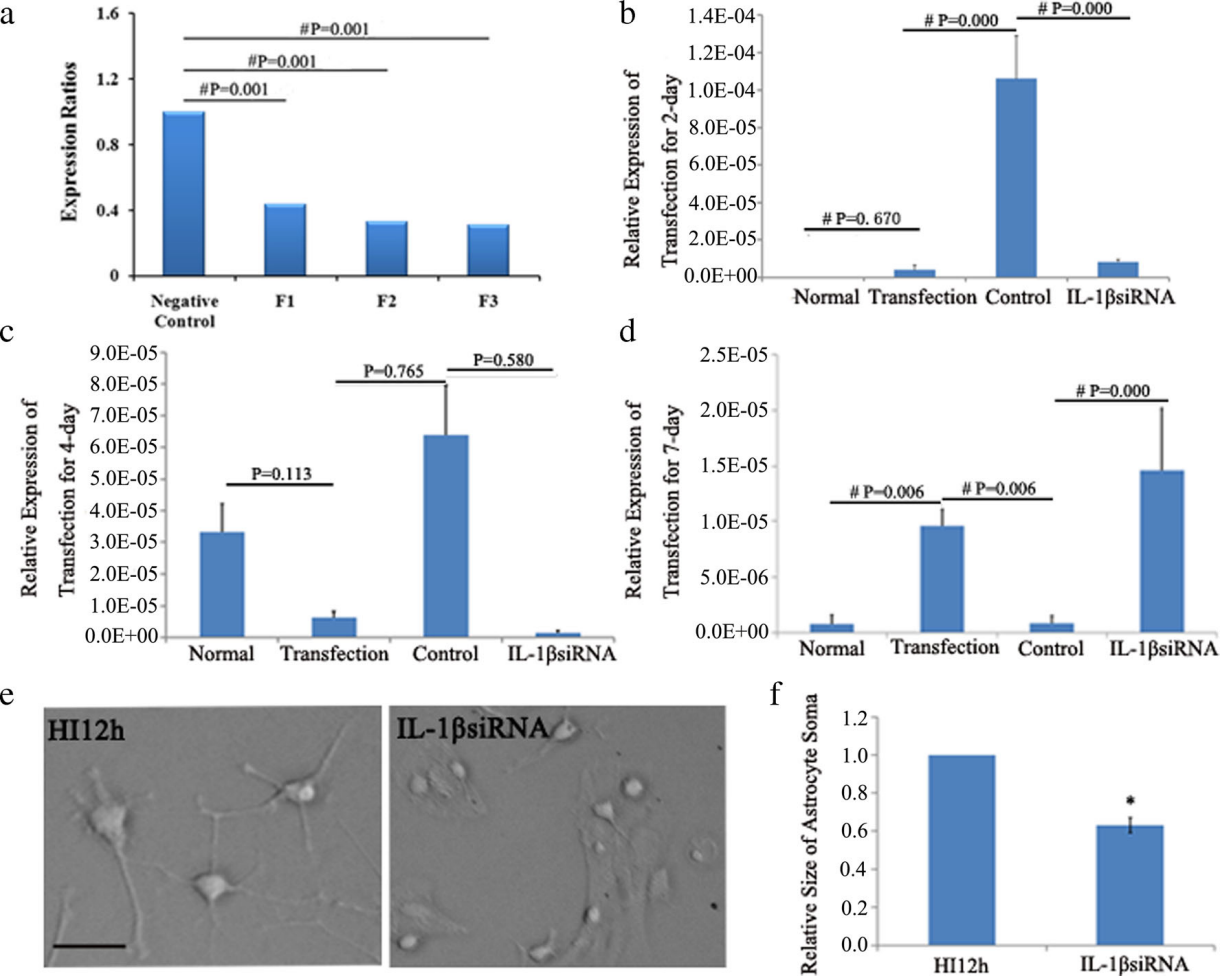
Cell swelling induced by HI was significantly attenuated at 2 days of transfection by F3 of IL-1βsiRNA. Inhibition rate of F3 was around 69 % compared to control one, while F1 and F2 that had an inhibition rate around 56 % and 67 % by real-time PCR analyses (**a**). After F3 transfection into primary astrocyte at 2 days (**b**), 4 days (**c**) and 7 days (**d**), real-time PCR analysis demonstrated the gene expression decreased significantly at 2 days after transfection, and no significance decrease was observed after 4 and 7 days (**b**–**d**). IL-1β down-regulation can significantly attenuate cell swelling induced by HI at 2 days transfection IL-1βsiRNA, compared with non-targeting siRNA (**e**). The quantification of the size of soma astrocyte is shown at **f**. Bar = 50 μm, shown in **e**. Statistical significance, *P < 0.05 and ^#^ *P* < 0.01

Three IL-1β-specific siRNA fragments (F1, F2, F3) were transfected into astrocyte for 2 days, and the silencing effect was examined by real-time PCR. Internalization into no-targeting fragment, the expression ratios of F1, F2 and F3 were 0.435, 0.330 and 0.308, respectively. Therefore, F3 has the maximum interference efficiency and was used in the following studies (Fig. 2a).

To explore the optimal siRNA-dependent silencing effect of IL-1β at 2, 4 and 7 days after transfection of F3, the mRNA levels were determined by real-time PCR, as described in Methods. Compared with the non-targeting sequence, IL-1β expression was most significantly inhibited after 2 days transfection (Fig. 2b–d). Therefore, F3 of IL-1βsiRNA and 2 days post transfection time point were used in the following studies.

### Effect of IL-1β siRNA on Astrocyte Morphology In Vitro

To explore the function of IL-1β, F3 of IL-1βsiRNA was transfected into astrocytes of HI for 2 days and the astrocyte morphologic changes were observed and collected by a phase contrast microscopy. Non-targeting siRNA was transfected into astrocytes as negative control. The size of soma of astrocyte showed marked decrease in IL-1βsiRNA injection group (*P* < 0.05 vs. HI12h) (Fig. 2e,f). These findings suggested that IL-1βsiRNA treatment can reverse swelling of astrocyte induced by HI.

### Functional and Morphological Consequence Induced by HI In Vivo

**Fig. 3:**
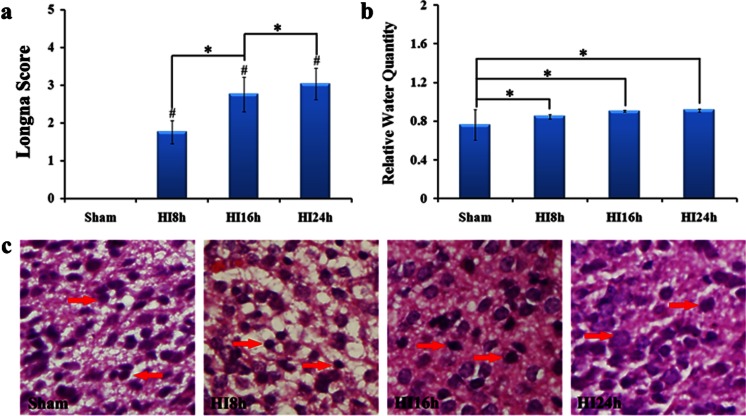
Changes of function and morphology induced by HI. **a** Longa score scale of sham group and HI group. Compared with sham group, HI can cause significant deficiencies at 8, 16 and 24 h (*n* = 12, *P < 0.05). Moreover, grading of severity of brain function was increased depending on time after HI (^#^ *P* < 0.05). **b** Brain tissue’s water quantity of sham group and HI group. Statistic analysis demonstrated that the water quantity of brain in HI rats exhibited a significant increase, compared with sham one. In addition, there was no significant difference between different time points of HI. **c** HE staining of sham group and HI group (×400; 8, 16 and 24 h). Morphologic result showed cells swelling and abnormally circular caused by HI in a time-dependent manner. Five fields were detected in each case. The *arrows* show the swelling cells and the polygons show the effusion of erythrocytes. Statistical significance, *P < 0.05 and ^#^ *P* < 0.01

Neural function following HI was assessed by Longa Score Scale, which was performed 8, 16 and 24 h after hypoxia (8 % O_2_, 92 % N_2_) until sacrifice. Statistic analysis showed that under HI intervention, Longa Score in the in HI groups (8, 16 and 24 h) was significantly increased compared with that of the control. Moreover, this score was gradually increased in a time-dependent manner with a statistically difference among different time points of HI groups (Fig. 3a). Water quantity of brain tissue started to dramatically increase after 8 h and gradually increased with time of HI treatment. However, there was no significant difference among HI groups (Fig. 3b). To investigate the effects of HI on brain edema, morphology changes were examined by HE staining. The result showed the ultrastructural changes of cells, and swelling cells with abnormally circular and enlarged space around cells were presented in HI group; while no abnormal signs were in normoxia group. Furthermore, basophil cell was more in HI group than that of in control group. Those results confirmed that brain edema with inflammation could be induced by HI (Fig. 3c).

### Effect of HI Event on Astrocyte Morphology In Vivo

**Fig. 4:**
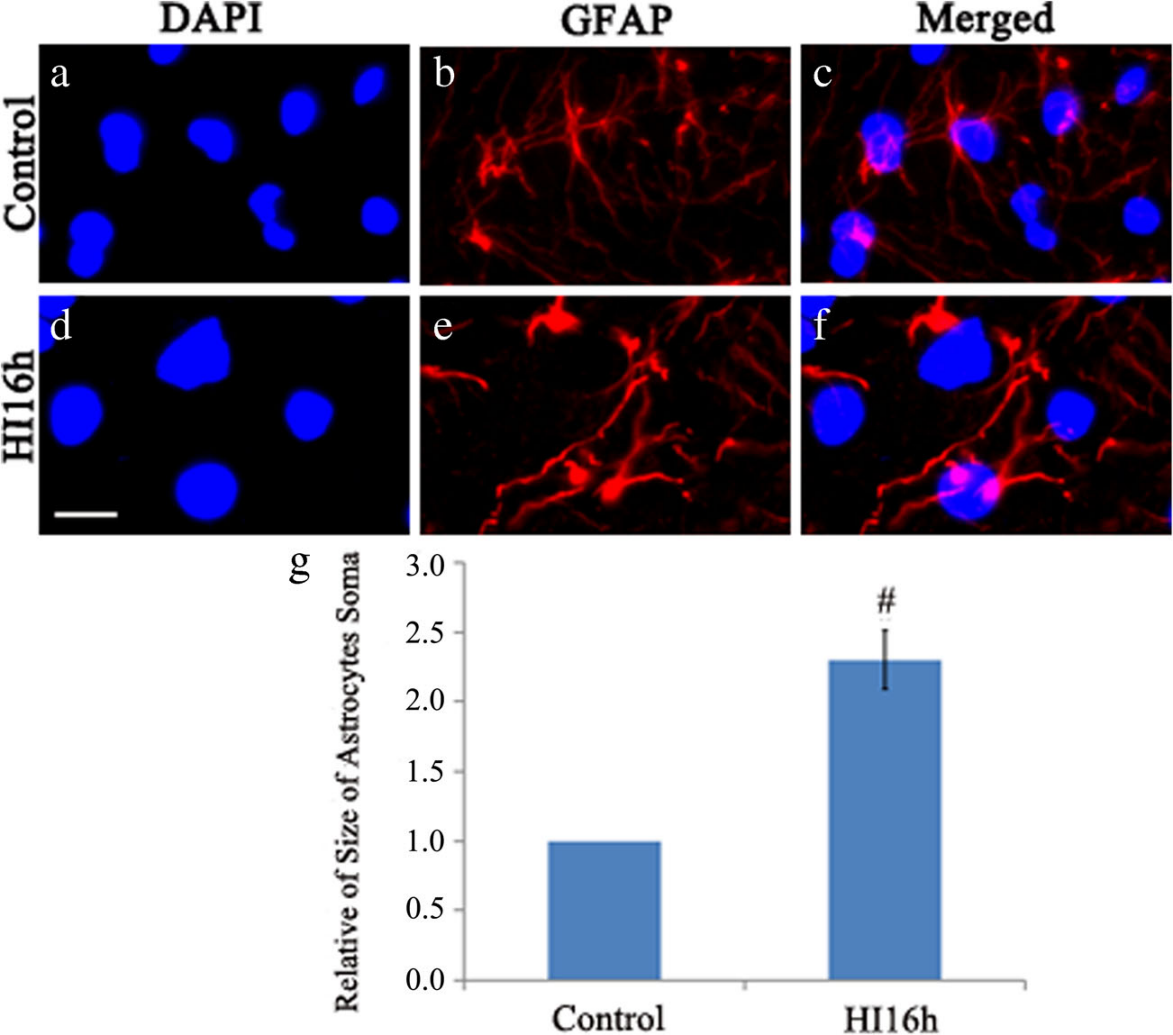
Effect of HI on astrocyte morphology in vivo. Immunofluorescent staining result showed that the size of soma of astrocyte was significantly increased following HI when compared with that in sham group. DAPI staining is presented in **a** and **d**. GFAP staining is presented in **b** and **e**. Merged image is presented in **c** and **f**. Quantitative analysis of soma is shown in **g** (*n* = 5, *P < 0.05). Bar = 50 μm, shown in **d**

Immunofluorence staining of GFAP was performed to determine the effect of HI on the morphology of astrocytes. The swelling GFAP positive cells were observed in HI group and the size of soma of GFAP positive cells was greatly increased (*P* < 0.01) when compared with that in the control group (Fig. 4), suggesting that astrocyte swelling was induced by HI administration.

### IL-1β mRNA and Protein Expression Change Following HI

**Fig. 5:**
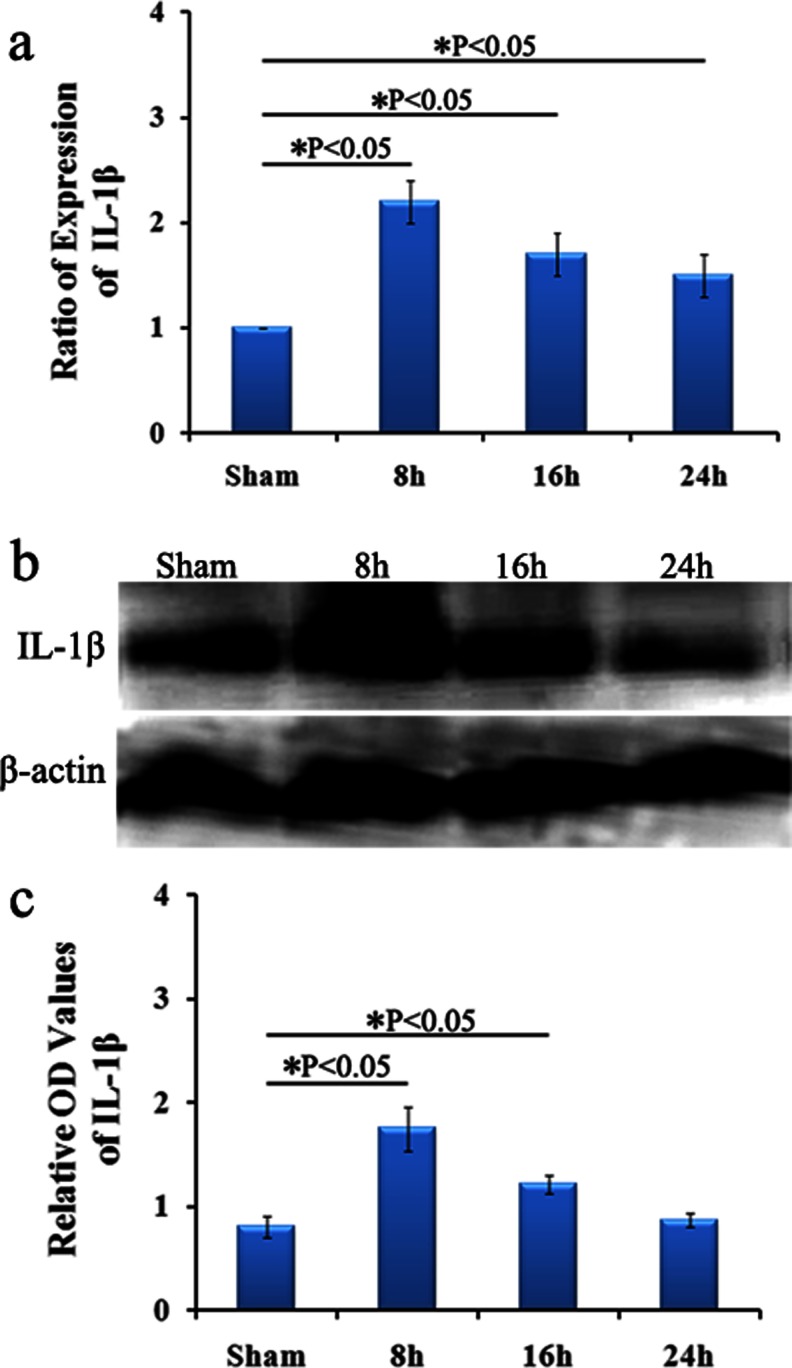
Changes of IL-1β expression following HI treatment. The mRNA expression of IL-1β is shown in **a**. Although no changes between different time points of HI groups were observed, there was a significant increase in the level of mRNA of IL-1β with 8, 16 and 24 h HI treatment (*P* < 0.05 vs. Sham). The protein expression of IL-1β is shown in **b** and **c**. Similar to the results of IL-1β mRNA expression, the expression of protein for IL-1β was significantly increased at 8 and 16 h after HI treatment when compared with the control (*P* < 0.05)

The effect of HI on the level of IL-1β mRNA and protein was measured by real-time PCR and Western Blotting analysis, respectively. IL-1β gene expression in brain tissue was increased remarkably in HI-treatment rats compared with that of rats maintained on the normoxia (Fig. 5a). As predicted, IL-1β protein expression showed a consistent pattern as mRNA levels detected by real-time PCR during HI treatment (Fig. 5b,c).

### Effect of Lentivirus Injection into Brain Tissue on IL-1β

**Fig. 6:**
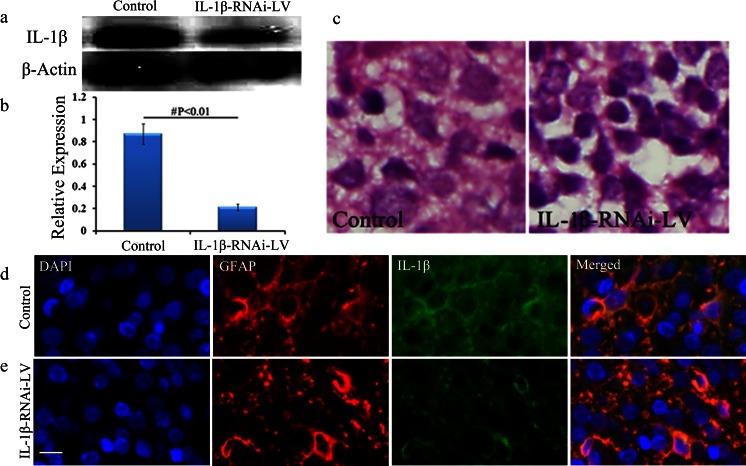
Effect of IL-1β-RNAi-LV injection into brain tissue. IL-1β protein was successfully silenced by lentivirus recombinant as shown by WB (**a**, **b**). Results of HE staining demonstrated IL-1β down-regulation by lentivirus can partially reverse brain edema induced by HI, when compared with negative control group. And swelling or necrosis and illegible borders of cell were ameliorated in the IL-1β-RNAi-LV group (**c**). **d**, **e** Co-localization of GFAP (*red*) and IL-1β (*green*) in astrocytes. The green fluorescence was significantly reduced in IL-1β-RNAi-LV group when compared with its control group, suggesting the level of IL-1β protein was successfully down-regulated by lentivirus. Moreover, the swelling of astrocytes induced by HI were partly reversed by IL-1β-RNAi-LV transfection. Bar = 50 μm, shown in **e**. ^#^Statistical significance of *P* < 0.01

After IL-1β-RNAi-LV injection into rats’ cortex, the protein expression of IL-1β was significantly reduced in IL-1β-RNAi-LV group (*P* < 0.01 vs. control; Fig. 6a,b). Moreover, immunofluorescent staining analysis of control and IL-1β-RNAi-LV groups showed IL-1β expression was down-regulated in IL-1β-RNAi-LV group (Fig. 6d,e). Those findings confirmed the fact that lentivirus recombinant of IL-1β undoubtedly silenced the protein expression.

### Astrocyte Swelling Ameliorated Following Lentivirus Injection and Co-localization of GFAP and IL-1β

After IL-1β-RNAi-LV injection into the rats’ cortex, immunofluorescent staining results showed the size of soma of GFAP positive cells (red) was diminished when compared with that one of control (Fig. 6d,e). Moreover, we found IL-1β^+^ cells (green) also expressed GFAP (red), suggesting IL-1β and GFAP were co-localized in astrocytes. Those results suggested astrocytes swelling resulted from HI was ameliorated after interference of IL-1β expression in brain tissue by lentivirus injection.

### Brain Edema Caused by HI Was Attenuated by IL-1β Interference

To examine the effect of IL-1β interference on brain edema caused by HI, HE staining was performed to assess the morphology changes of the brain tissue. Compared with control group, IL-1β interference reduced brain defects induced by HI, including swelling or necrosis and illegible borders of cell (Fig. 6c).

### IL-1β Regulates Inflammation Factors Expression

**Fig. 7:**
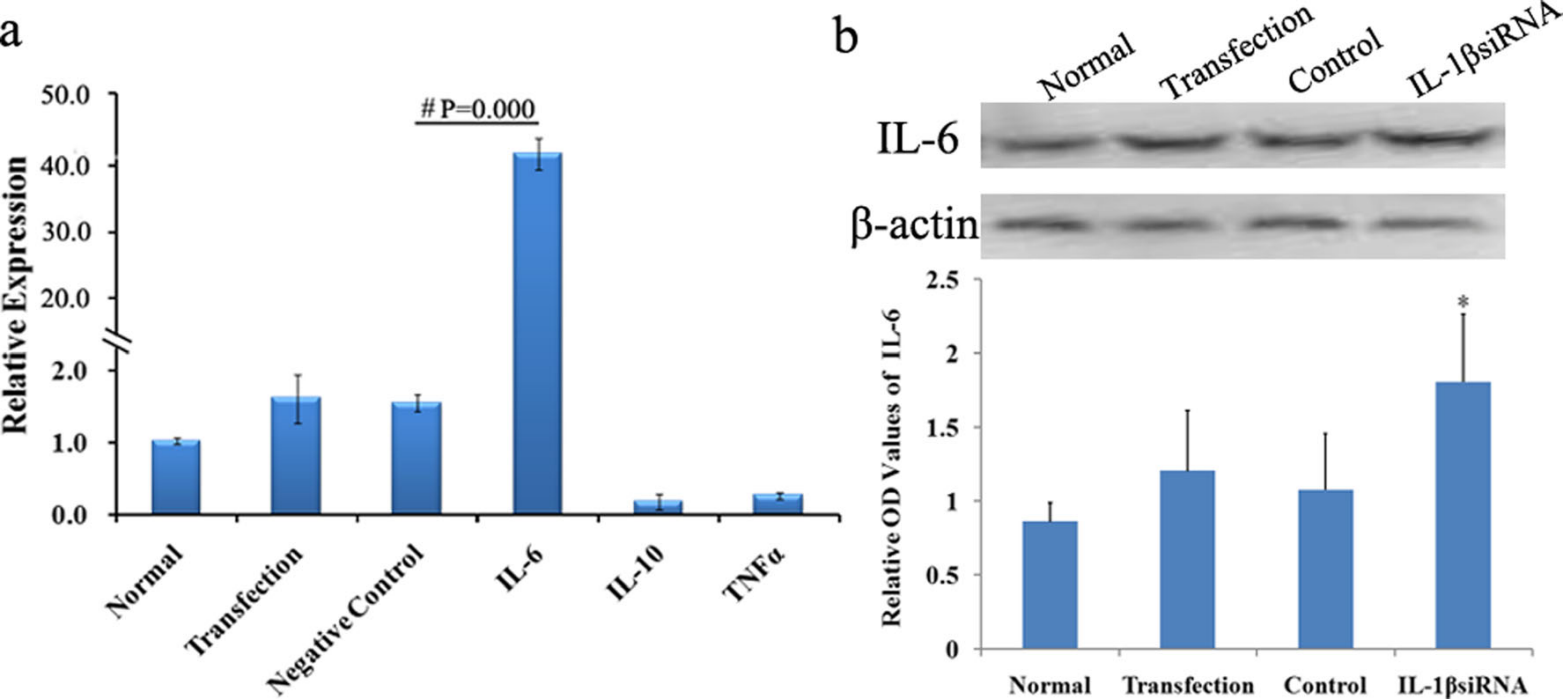
Inflammation factors expression regulation by IL-1β. Compared with negative control, although no statistically significant difference was observed of IL-10 and TNF-α expression, IL-6 gene expression was significantly increased after down-regulation of IL-1β (**a**). And IL-1β protein expression was also significantly increased after HI treatment when compared with the control (b). ^#^Statistical significance of *P* < 0.01

To further explore the downstream signaling molecules of IL1-β and underlying mechanisms of IL-1β interference induced beneficial effect on HI, real-time PCR was performed to examine the changes of IL-6, IL-10 and TNF-α expression after IL-1β interference. Compared with negative control group, the mRNA expression of IL-6 was significantly increased (*P* = 0.000; Fig. 7a), while no significant effect on IL-10 and TNF-α gene expressions. Consistent with the mRNA expression change, the protein expression of IL-6 also showed increase after IL-1β down-regulation (Fig. 7b).

## Discussion

This study demonstrated that neurologic deficit, brain edema and astrocyte swelling were caused by HI, which was accompanied by IL-1β up-regulation. However, this pathological phenomenon was reversed and attenuated after IL-1β down-regulation by siRNA in vitro and lentivirus in vivo. And our findings suggested that IL-6 may be the downstream signaling molecule of the IL-1β and associated with regulation of HI.

In the present study, the primary culture astrocyte was administrated free-serum medium and exposed 2 % O_2_ and 98 % N_2_ for 6, 12 and 18 h to establish cell model of HI. Three days postnatal rats were subjected the right carotid artery ligation and subsequently exposed to 8 % oxygen for 8, 16 and 24 h, respectively, to establish animal model of HI. We examined the variation of morphological and functional of astrocyte and brain tissue following HI, and cellular swelling, inflammatory cell infiltration and cellular necrosis were observed and margin of cells was not clear in HI group; the degree of neural dysfunction was present in time-dependent manner under HI intervention; the water quantity of brain tissue also gradually increased with the extent of time of HI. Given that those changes had a statistical significance when compared with their control group, we reasoned that an HI model was successfully established.

In this study, IL-1β was increased significantly in mRNA level by real-time PCR following HI administration. The results of recent studies were confirmed by our study. For instance, up-regulation of IL-1β, ROS, and IL-8 by hypoxia/ischemia occurred in human chondrocytes [11], similar to the finding in HI of newborn rats that demonstrated the expression of proinflammatory cytokines (TNF-α, IL-1β, IL-10) was increased prominently 3 h after HI compared to mRNA levels in sham-operated animals [13]. Conversely, Savman et al. [16] suggested IL-1β had no relationship with the consequence of HI, but was only observed in the most severe cases of infants who eventually die or develop serious disabilities. This may be due to the different target tissue examined. Difference from vasogenic brain edema, cytotoxic brain edema induced-HI has intact blood–brain barrier (BBB) [17]. Such an interesting discrepant finding may indicate that the source of increased IL-1β in the brain tissue is due to local cellular production of central nervous system as opposed to overflowing from serum via BBB. This explains why IL-1β in the serum is not correlated with the outcomes of HI in infants in other studies.

To explore the role of IL-1β in HI encephalopathy, we designed siRNA and constructed the lentevirus vector of IL-1β shRNA to interfere its expression. The maximum interference rate of siRNA fragment and the optimization time point of transfection were selected in vitro. Considering IL-1β was richly expressed in astrocytes suffered from HI, which was supported by the result of fluorescence double-labeling of GFAP and IL-1β, astrocyte was cultured as a good target cell for transfection. Compared to negative controls, F3 of siRNA-IL-1β had the maximum interference efficiency, and 2 days of transfection was the optimization time point. Based on those findings, we employed F3 (CTGCAGGCTTCGAGATGAA) for the following construction lentevirus experiments.

After microinjection the lentivirus in the cortex motor area of HI rat, Western blot and immunofluorescent staining results showed IL-1β protein level was down-regulated significantly in IL-1β-RNAi-LV group when compared with that in control group. Moreover, the brain edema, swelling astrocyte and neurologic deficit following HI were attenuated with down-regulation IL-1β. Consistent with our findings, several studies have also shown that there was a beneficial neuroprotective role during down-regulation of IL-1, including inhibition of microglial activation, neutrophil infiltration, and cytokine levels. IL-1 receptor antagonist (IL-1ra), a naturally occurring protein, binds to IL-1 receptors and blocks several actions of IL-1. IL-1ra in the brain was reported as an endogenous neuroprotective molecule in 1997 [18]. The volume of brain infarct and disruption of BBB in ischemic brain of rat were significantly reduced after administration of IL-1ra [19]. Similarly, IL-1ra was used for patients with subarachnoid hemorrhage (SAH) to reduce brain injury induced by focal cerebral ischemia in clinical therapy [20]. Upon analysis, we blocked the actions of IL-1β by injection IL-1β-RNAi-LV in our study, similar with the administration of IL-1ra.

Further study was performed to explore the molecular mechanism of beneficial effects of down-regulation of IL-1β on HI. We the ex found that IL-6 was significantly increased when IL-1β expression was reduced.

These findings suggest that IL-1β may be a target gene therapy of HI brain edema in neonates. The method of down-regulation of IL-1β by RNA interference during HI may protect against drain edema, stoke and other brain disorders via up-regulation of IL-6 expression.
